# Enrolling Adolescents in Research on HIV and Other Sensitive Issues: Lessons from South Africa

**DOI:** 10.1371/journal.pmed.0030180

**Published:** 2006-04-18

**Authors:** Jerome Amir Singh, Salim S Abdool Karim, Quarraisha Abdool Karim, Koleka Mlisana, Carolyn Williamson, Clive Gray, Michelle Govender, Andrew Gray

## Abstract

Jerome Singh and colleagues present the ethical and legal challenges surrounding the enrollment of adolescents in HIV observational studies and other types of sensitive research.

With more than 6 million HIV positive individuals—the highest of any country in the world—South Africa is the epicentre of the HIV/AIDS pandemic. In much of sub-Saharan Africa, especially South Africa, the HIV/AIDS epidemic is growing fastest in teenagers, especially young girls (see sidebar) [1]. Hence, any future vaccination strategy in this setting will, of necessity, have to target young adolescents prior to sexual debut.

The current candidate HIV vaccines that are being considered for efficacy trials are likely to act principally by reducing viral loads, hence reducing both transmission to sexual partners and progression to AIDS [2]. One of the limitations to conducting HIV vaccine trials is the dearth of data on important HIV disease parameters such as viral load, immune responses, and disease progression in African adolescents. While there is observational research currently being conducted in the United States on adolescents, such as the Reaching for Excellence in Adolescent Care and Health (REACH) study [3], there is a need for information on set point, HIV transmission, and disease progression in adolescents from Africa, even if only to confirm whether the disease process in African adolescents differs from those in developed countries.

The Joint United Nations Programme on HIV/AIDS notes that children, including infants and adolescents, should be eligible for enrolment in HIV preventive vaccine trials, both as a matter of equity and because young adolescents and children are at high risk of HIV infection [4]. However, no distinction is drawn between adolescent participation with parental consent and the autonomous participation of adolescents. This distinction is particularly important in sub-Saharan Africa, where it is often difficult or impossible to obtain parental consent.

The Centre for the AIDS Programme of Research in South Africa (CAPRISA), which is funded by the US National Institutes of Health, recently proposed a follow-up study of adolescents and young adults with acute HIV infection to obtain the essential information on subtype C viral set point, prognostic viral load measurements, and disease outcome—all important biological outcome markers in HIV vaccine efficacy trials. The study proposed to enrol, as one of its groups, adolescents as young as 14 years old from a prenatal and family planning clinic, where those who volunteered could do so without parental consent when parents or legal guardians were not available. Three ethics committees supported the enrolment of this cohort, although a fourth did not. As a result, the study protocol was amended to include participants only above the age of 18.

Several works have highlighted the ethico-legal challenges implicit in enrolling adolescents in South African HIV vaccine trials [5–7]. However, the enrolment of autonomous adolescents in crucial HIV observational studies and in other types of sensitive non-HIV research in South Africa is equally problematic. We present the ethico-legal challenges, as well as the scientific and social grounds, to justify the autonomous participation of adolescents in HIV observational studies—such as the CAPRISA study—as well as in future HIV vaccine trials in South Africa and much of the rest of sub-Saharan Africa. We argue that the same reasoning applies to other types of sensitive non-HIV research involving adolescents. We also suggest how conflicts between restrictive laws and flexible ethics guidelines should be addressed.

## Social Considerations: Obstacles to Parental Consent

The Human Sciences Research Council reports an almost 2-fold increase in South African households headed by children or consisting only of children (referred to as “child-headed households”, i.e., orphans or children without resident adult guardians) between 2002 [8] and 2005 [9]. In the 2005 study, among children 12–18 years of age, 2.6% (or 180,433) identified themselves as heads of households, with the majority being African. Among orphans 12–18 years of age, 2.8% (or 213,859) identified themselves as being heads of households. The 2005 Human Sciences Research Council study also highlighted statistics that indicate that almost 65% of orphans live in community or family care, or child-headed households.

Our data supports these findings and show that many of the adolescents attending the prenatal and family planning clinics do not live with their parents [10]. Some live alone, either because their parents are deceased or because they work and live in a distant urban area. Others have been left in the care of members of the community (usually relatives). Most of these surrogate caregivers are not formally appointed or recognised as the child's legal guardian. In these instances, it is practically impossible to seek parental consent or to determine who, if anyone, is the legal guardian to authorise an adolescent's participation in a study. [Fig pmed-0030180-g001]

**Figure pmed-0030180-g001:**
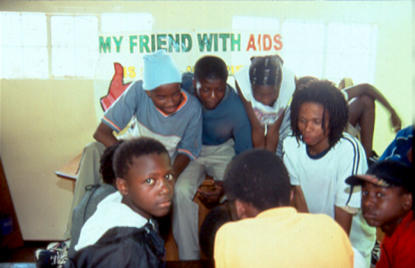
Sex education at an adolescent drama group in Soweto, South Africa (Photo: Arjen van de Merwe/Population Concern)

## Ethical Considerations

According to the Council for the International Organisation of Medical Sciences Guidelines, “In some jurisdictions, individuals who are below the general age of consent are regarded as ‘emancipated’ or ‘mature’ minors and are authorized to consent without the agreement or even the awareness of their parents or guardians” (see commentary on guideline 14 in [11]).

Prenatal and family planning clinic attendees are often selected for enrolment into HIV studies because they are sexually active, often times have multiple partners, and are already familiar with clinical procedures. Given the observations on child-headed households above, there are good grounds to consider such adolescents as “mature minors”, as this recognises the social reality of the study population. Acknowledgment of this reality makes it unnecessary to seek supplemental parental consent for adolescent participation in studies.

On the issue of mature minors and the need to obtain additional parental informed consent, the Council for the International Organisation of Medical Sciences Guidelines state the following: “Some studies involve investigation of adolescents' beliefs and behaviour regarding sexuality or use of recreational drugs; other research addresses domestic violence or child abuse. For studies on these topics, ethical review committees may waive parental permission if, for example, parental knowledge of the subject matter may place the adolescents at some risk of questioning or even intimidation by their parents” (see commentary on guideline 14 in [11]).

An HIV-positive test result often carries a negative connotation and stigma. Discrimination, violence, and social ostracism are often experienced by those, especially women, who disclose their HIV status to partners and family members. There is also the concern that adolescents are not willing to participate if their HIV status will be disclosed to their parents. In such cases, parents may want to be informed of their children's HIV status by researchers and health-care providers even if it is against their children's wishes, thereby violating the study participant's right to confidentiality. As a serious consequence of violating this right, participants could be inadvertently deterred from seeking necessary treatment, a decision that would have adverse consequences for their health.

The researcher's willingness to respect an adolescent participant's autonomy and right to confidentiality will likely determine that adolescent individual's willingness to participate in research. Adolescent participants would enrol in such studies on the legitimate expectation that their confidentiality would be respected. Requiring these participants to seek parental consent for their participation in the study would effectively nullify this obligation to assure confidentiality. Moreover, researchers or health-care providers who breach the adolescent participant's confidentiality to parents could violate section 14 of South Africa's Constitution [12], which entitles everyone (including minors) to the right of privacy.

In some countries, such as the US, ethical review committees have the authority to waive a requirement for parental permission for adolescent participation when there are compelling reasons warranting this action. Yet, despite the merits of doing so, ethical review committees in South Africa have been hesitant, not only for adolescents of 14 years of age, but even for adolescents of 16 and 17 years of age. This is compounded by the current ethico-legal position in South Africa, where the current South African Medical Research Council (MRC) research ethics guidelines (General Principles) prescribe 14 years of age as the autonomous age of consent for therapeutic research but not observational studies [13]. The General Principles stipulate that therapeutic research may be undertaken with the independent consent of a minor over the age of 14. It regards the pursuit of additional parental consent in such instances as “desirable” but not mandatory. On the issue of nontherapeutic research (including observational research of a nontherapeutic nature, such as the CAPRISA study, which may involve invasive procedures such as blood draws), the General Principles provide that such research in the context of adolescents is generally not permissible, except where parental consent (and the assent of the minor concerned) is obtained. The General Principles state that “…where the research is of such a nature that there is no possibility of harm to the child, either physically or psychologically, the minor may consent independently, provided he is intellectually of sufficient maturity to understand the nature of the procedure and to give voluntary, informed consent. As a general rule it may be assumed that a child below the age of 14 is not of such maturity” [14].

The General Principles also contain many other flaws and inconsistencies in regard to research on adolescents [15]. In short, the General Principles are counter to the interests of adolescents. They fail to acknowledge that the acquisition of additional parental or guardian consent would, in some instances, (1) be logistically impossible, (2) necessitate breaching the confidentiality of research participants, and (3) place the participant at potential risk of harm. These concerns also apply to adolescents involved in sensitive non-HIV-related research. Although the MRC has issued more flexible ethics guidelines on HIV vaccine research [14], these guidelines do not apply to research sponsored by agencies other than the MRC, and they are not applicable to observational research. While South Africa's Good Clinical Practice Guidelines [16] also offer guidance on adolescent research, they, too, do not govern observational research. So how can following the General Principles affect HIV observational studies?

Our work in a rural part of South Africa has revealed that adolescents, some as young as 12 years old, routinely seek antenatal care, treatment for sexually transmitted infections, or family planning at district clinics. They do so autonomously, despite the legal age for consent to treatment being 14 years of age, and, perhaps, they do so because they do not have parents or legal guardians or because they perceive that confidentiality is assured. These factors no doubt motivate adolescents to present at clinics for treatment in other settings, too. Following the guidance of the General Principles for adolescent research attached to such clinics will make such research impossible. Although the General Principles are, strictly speaking, only binding on MRC-sponsored studies, they are generally considered influential among members of South Africa's research ethics community.

Adolescents' best interests will be served by their autonomous participation.

Matters will be compounded when the country's newly promulgated National Health Act (NHA) becomes operational. The NHA mandates the solicitation of parental consent for adolescent enrolment in therapeutic and nontherapeutic studies, as well as ministerial consent in the case of nontherapeutic studies. The NHA does not prescribe definitions for the terms “child” and “minor”, although it uses both terms interchangeably in relation to both types of studies, which is problematic since there is no uniform definition for these terms in South African law [17].

In May 2005, South Africa issued its first national health research ethics guidelines intended to govern all health-related research in the country. Entitled Ethics in Health Research: Principles, Structures and Processes (EHR), the document describes itself as the country's “national policy on the ethical practice of research”, and makes explicitly clear that its principles should guide “all research involving animals and human participants in any discipline relating to health” [18]. The EHR is intended to be read in conjunction with the relevant provisions in the NHA that govern research. However, some of its provisions are incompatible with the NHA.

Paragraph 5.1 of the EHR governs research on minors. It begins by declaring that minors should participate in research “only where their participation is indispensable to the research and where participation is not contrary to the individual minor's best interests”. In terms of the EHR, “child” is taken to mean a person who has not yet reached puberty, while “adolescent” means a person who has reached puberty. The EHR's glossary section, which bases its definitions on the Canadian Code of Ethical Conduct for Research Involving Humans (1996) and the International Conference on Harmonisation Guidelines for Good Clinical Practice, defines “child” slightly differently. It provides that a child “is a minor who lacks the legal ability to make a decision whether or not to participate in research”. Despite its explicit reference to the law, the EHR, like the NHA, steers clear of providing a legal definition of the term “minor”. However, unlike the NHA, which does not recognise the rights of children to autonomously participate in research in any circumstance, the EHR adopts a flexible approach that seemingly puts it at odds with the NHA. It recognises that “adolescents may be capable of consenting themselves to certain types of research participation and that, for particular types of research, it may be desirable that they do so unassisted”. In this regard, the EHR can be said to be more responsive to South Africa's increasing social reality of “mature minors” and child-headed households.

The EHR also seems to have heeded the concerns of investigators participating in sensitive research involving adolescents, who voiced their concerns about the inflexibility of the NHA on this issue. Paragraph 5.3.1 of the EHR accordingly advises research ethics committees (RECs) that research involving adolescents who may consent unassisted should be approved only if (1) the research, including observational research, places the adolescent at no more than minimal risk, and only if (2) the nature of the research is such that, in the opinion of the REC, the parents or legal guardians or community at large are unlikely to object to the adolescent autonomously consenting to participation in the investigation. The opinion of the REC must be informed by information gathered from the community concerned and by contributions from the lay members of the committee.

“Minimal risk research”, according to the EHR, is that which “anticipates that the probability and magnitude of harm or discomfort to be experienced in the research will not be greater than those ordinarily encountered in daily life”. This resonates with the understanding of the term in the American Code of Federal Regulations (45 CFR 46.102). The EHR stipulates that in all cases, the protocol must provide sufficient information to justify clearly why adolescents should be included as participants and must justify clearly why the adolescent participants should consent unassisted.

## Conflicts between the Guidelines and the Law: How Should Ethics Committees Respond?

Given that the NHA and EHR take different stances on the autonomous participation of children in research, how should RECs deal with this apparent conflict? Conventional wisdom dictates that provisions of law take precedent over provisions in policy or guidance documents. However, there are at least five important reasons why a REC should embrace the stance of the EHR, in regards to research on minors, over that of the NHA, notwithstanding the latter's legal nature.

First, adhering to the flexible stance of the EHR will make it possible to investigate valuable and necessary sexuality-related research on adolescents, of the type outlined above.

Second, a REC's primary mandate should be to make decisions based on ethical considerations versus exclusively legal considerations. Blindly following ill-considered law could evidence unethical and unconstitutional outcomes.

Third, the EHR was launched by the minister of health in the aftermath of the promulgation of the NHA and the numerous concerns that were raised about its provisions pertaining to research on minors. It should be assumed that the minister applied her mind to the issue, realised the inconsistency between the NHA and the EHR, but gave her unreserved endorsement to the latter because doing so was in the best interests of adolescent minors. To assume otherwise would imply that the minister did not apply her mind to the implications of the EHR before approving it.

Fourth, as in other countries, South African law holds that the best interests of the child must dictate what approach to follow in matters pertaining to a child. Given the invaluable biological, sociological, behavioural, and clinical data that the inclusion of an adolescent cohort will generate in HIV studies, the enrolment of this cohort is crucial for future prevention and treatment interventions in this group. Accordingly, the best interests of adolescents will be served by their autonomous participation in such studies. The NHA is intended to protect the interests of children. RECs should respect the spirit of the law rather than merely the letter of the law. The interests of children will be protected and promoted by their participation in minimal risk HIV observational studies.

Fifth, in growing recognition of the autonomous decision-making abilities of young individuals, South Africa's draft Children's Bill [19] promises to bring sweeping changes to the law in regards to children. The bill, several years in the making, is the most significant postapartheid legal instrument governing the affairs of children. Upon promulgation, the bill will repeal several key statutes that currently govern the affairs of children, including the Children's Act, (Act 33 of 1960), the Age of Majority Act (Act 57 of 1972), and the Child Care Act (Act 74 of 1983). Part 3 of the bill is explicitly dedicated to matters relating to the health of children. Section 129 of the bill governs consent to medical treatment of or surgical operation on a child. In a break with current law, the bill lowers the age of consent for medical treatment. It provides that children may consent to their own medical treatment (or to the medical treatment of their child) if over the age of 12 years, of sufficient maturity, and with the mental capacity to understand the benefits, risks, and social and other implications of the outcome. Similarly, the bill also provides that children may consent to the performance of a surgical operation on themselves (or their child) if over the age of 12 years, of sufficient maturity, and with the mental capacity to understand the benefits, risks, and social and other implications of the surgical operation, and where duly assisted by their parent or guardian. Admittedly, the bill does not pertain to research. However, the Children's Bill is a more recent law than the NHA, and it overwhelmingly demonstrates that the South African legislature explicitly recognises that adolescents as young as 12 years of age are capable of making important decisions autonomously, a factor that RECs should note.

## A Proposal for the Way Forward

As adolescents are bearing the brunt of the AIDS epidemic in much of sub-Saharan Africa, we believe studies in this group are critical. We have argued that it is ethically justifiable to enrol adolescents in certain cases without seeking parental consent for adolescent participation in HIV research. The same might apply to certain types of sensitive non-HIV research involving adolescents who could be compromised if parental consent was made mandatory (for example, research on child abuse, teenage pregnancy, and teenage sexuality). We accordingly encourage local RECs to exercise their discretion to authorise the waiver of additional parental consent in studies involving adolescents on a case-by-case basis.

The research regulatory framework of the US—which is much more comprehensive than that of South Africa or the rest of sub-Saharan Africa—could prove instructive. The American Code of Federal Regulations (45 CFR 46.408c) endorses the waiver of parental consent in instances where the pursuit of consent could be detrimental to the minor (as we have argued is applicable in South Africa). Ethical review committees should also note that regardless of South Africa's lack of uniformity on the legal age of majority, the World Health Organization Guidelines for Research on Reproductive Health Involving Adolescents [20] states that there is no clear ethical justification for excluding from research adolescent individuals “below the age of legal majority”, and that an ethical duty of beneficence and justice might exist to conduct appropriate research on this group. We strongly urge ethical review committees to consider these persuasive factors in pondering the merits of permitting autonomous adolescent participation in all research on a case-by-case basis.

While it is difficult to establish truly representative community structures for consultation on research issues, the approach of engaging with the communities where the research is being conducted could be one part of the solution [21,22]. This could be done through existing community representative bodies or through the establishment of a representative community structure from the intended study population [23]. This creates the opportunity to discuss the rationale underpinning the inclusion of adolescents in the research, as well as the reasons for not making parental consent for an adolescent's participation in a study a requirement. We believe that the social reality of child-headed households, as well as community endorsement of this approach, should factor strongly into a research ethical review committee's consideration in its deliberations on whether to allow adolescents to provide autonomous consent for participation in a study.

## Conclusion

We do not believe that the challenges that face South African researchers in enrolling adolescent participants in HIV studies, such as the CAPRISA study, are unique to the country. The challenges probably apply to much of sub-Saharan Africa, where future vaccine and microbicide efficacy trials will need to be conducted if we are to fast track efforts to find a vaccine or microbicide to reduce the spread of HIV in this important population. Moreover, we also believe that the factors we argued for in favour of the autonomous participation of adolescents in HIV-related studies apply equally to certain types of sensitive non-HIV research. We recommend that investigators involved in such research endeavours urge the ethics committees that oversee their research to embrace the stance of the EHR over that of the NHA for the reasons we outlined above.

The need to protect adolescents from harm in research needs to be carefully balanced with the need to undertake research in this population to find solutions to this epidemic. To this end, rigid legislation and/or ethical guidelines that pertain to adolescent participation in research and their uncritical application are counterproductive. We need to be cognizant of this inherent conflict and create an enabling ethico-legal framework to avoid inadvertently doing more harm than good to the intended study population.

**The Critical Need to Study Young Adolescent Women**

The rapid spread of HIV among young women, and particularly among adolescent girls in South Africa, has been described as “explosive” [1]. Year 2005 estimates of HIV incidence among South African youth aged 15–24 years is 3.3% [9]. Alarmingly, females in this age group have a five-times-higher HIV incidence than males (6.1% versus 0.8%) [9]. In addition to the public health imperative to reduce HIV risk in this age group, a deeper understanding of the factors influencing HIV risk in adolescent girls is critical for altering the nature and course of the epidemic in sub-Saharan Africa.

In one rural South African district, the prevalence of HIV infection in pregnant women increased from 26% in 2001 to 34% in 2002 [8]. Importantly, 39% of the pregnant women in this district were younger than 19 years of age, with the youngest being 12 years of age. In pregnant women younger than 19 years of age, the HIV prevalence was 25.8%, and the incidence rate is estimated to be as high as 9.6% per annum. HIV prevalence among girls younger than 19 years attending family planning clinics in this district was 27.5% in 2002. The sheer scale of the epidemic in this one rural district highlights the critical need for HIV prevention, treatment, and research in adolescents.

## Abbreviations

CAPRISA: Centre for the AIDS Programme of Research in South Africa
EHR: Ethics in Health Research: Principles
MRC: South African Medical Research Council
NHA: National Health Act
REC: Research Ethics Committee
